# The impact of COVID-19 on emergency surgical presentations in a university teaching hospital

**DOI:** 10.1007/s11845-021-02709-w

**Published:** 2021-07-12

**Authors:** Ahmed M. Kamil, Matthew G. Davey, Fadi Marzouk, Rish Sehgal, Amy L. Fowler, Kevin Corless, Maeve O’Neill, Chris G. Collins

**Affiliations:** 1 Department of Surgery, Galway University Hospitals, Galway, Republic of Ireland; 2 Department of Academic Surgery, National University of Ireland, Galway, Republic of Ireland

**Keywords:** COVID-19, Emergency surgery, Patient outcomes, Surgery

## Abstract

**Introduction:**

The Coronavirus-19 (COVID-19) pandemic has led to a 50–70% reduction in acute non-COVID-19 presentations to emergency departments globally.

**Aim:**

To determine the impact of COVID-19 on incidence, severity, and outcomes of acute surgical admissions in an Irish University teaching hospital.

**Methods:**

Descriptive data concerning patients presenting with acute appendicitis, diverticulitis, and cholecystitis were analysed and compared from March–May 2020 to March–May 2019.

**Results:**

Acute surgical admissions decreased in March from 191 (2020) to 55 (2019) (55%), before increasing by 28% in April (2019: 119, 2020: 153). Admissions due to acute cholecystitis reduced by 33% (2019: 33, 2020: 22), with increased severity at presentation (*P* = 0.079) and higher 30-day readmission rates (*P* = 0.056) reported. Acute appendicitis presentations decreased by 44% (2019: 78, 2020: 43, *P* = 0.019), with an increase in severity (*P* < 0.001), conservative management (*P* < 0.001), and post-operative complications (*P* = 0.029) in 2020 compared to the same period in 2019.

**Conclusion:**

COVID-19 has potentiated a significant reduction in acute surgical presentations to our hospital. Patients presenting with acute appendicitis during the pandemic had more severe disease, were more likely to have complications, and were significantly more likely to be managed conservatively when compared to historical data.

## Introduction

In December 2019, an increase in the number of cases of viral pneumonia complicated with acute respiratory distress syndrome was observed in Wuhan, China. The Severe Acute Respiratory Syndrome Coronavirus (SARS-CoV-2 or COVID-19) viral infection spread exponentially worldwide, leading to an international public health emergency. [1].

In the forewarned countries of the western world, patients initially avoided presenting to hospitals due to fear of COVID-19 and its devastating effects [2]. Consequentially, global data depicted reduced numbers of people seeking medical advice for acute conditions and illness, and a delay in presentations [3–6]. The shift in this clinical paradigm has had significant effects on patient outcomes worldwide, as is evident in data published in several international studies. A large European series from Spain detailed a 40% reduction in catheterisations being performed for ST-elevation myocardial infarction across 81 institutions, while another series recorded a 20% decrease in presentations with myocardial infarction in centres in the USA [2, 5]. Other authors describe a series including 12 delayed acute paediatric presentations due to COVID-19 across five Italian hospitals in Northern Italy. They observed six children requiring urgent intensive care admissions, four of whom suffered mortality as a consequence [4]. Furthermore, there have been reports of a four-fold increase in time to first contact with medical professionals in Queen Mary Hospital, Hong Kong, since the outbreak of the pandemic [6].

These studies highlight the implications that COVID-19 has had on emergency services worldwide; however, there is limited data focusing on COVID-19 and acute emergency surgical presentations. The aim of the current study was to quantitatively analyse differences in presentation, management, and outcomes of common acute surgical conditions during the COVID-19 pandemic, compared with historical data one year previously.

## Methods

Local ethical approval was obtained from the clinical ethical research council (Ref: C.A 2449). A retrospective cohort study was conducted at a tertiary referral hospital (Galway University Hospital, Ireland) between 1 March and 31 May 2020 and compared to the same time period 1 year previously (1 March to 31 May 2019). Patients presenting with acute appendicitis, acute diverticulitis, and cholecystitis were the main focus of the study.

Clinical, laboratory, and radiologic data concerning these presentations were collected using Emergency Department (ED) and Acute Surgical Assessment Unit (ASAU) logbooks, paper and electronic chart review, hospital electronic patient enquiry software, and electronic discharge summary. Inclusion criteria were all patients admitted acutely under a general surgical team with (1) acute appendicitis, (2) acute diverticular disease, or (3) acute gallbladder pathology (acute cholecystitis including cholangitis) within the specified time periods above. Data points collected include patients demographics, duration of symptoms, surgical pathology type, treatment method (surgical vs conservative), post-operative complications (Clavien-Dindo classification) [7], total length of hospital stay (LOS), 30-day readmission rate, and reason for readmission. Moreover, failure of conservative management was recorded, defined as no clinical improvement, and/or deterioration at 48 h from admission.

The simplified acute physiology score (SAPS-II) was used for all patients in the study to compare overall severity at presentation [8]. Tokyo Guidelines 18/13 system was used to assess severity of disease in gallbladder pathology [9]. For patients with acute appendicitis, two different scoring systems were used: the radiological appendicitis severity index (APSI), based on radiological assessment of appendicitis, as well as the disease severity score system (DSS) which assessed disease severity based upon histology and intraoperative findings [10, 11]. The Hinchey classification system was used for categorizing the severity of illness in the diverticulitis group [12].

All data were analysed using descriptive and inferrential statistics; Fishers exact, Chi-Squared, independent Student’s t, and one-way analysis of variance (ANOVA) tests were used as appropriate. All tests of significance were 2-tailed, with *P* < 0.05 indicating statistical significance. Data was analysed using Statistical Package for Social Sciences™ (SPSS™) version 26.0.

## Results

### Clinical characteristics

Eight hundred and fifty nine patients presented with an acute surgical emergency during the course of this study. There was a 10.4% decrease in acute surgical presentations overall (453 in 2019 versus 406 in 2020). One hundred and thirty-eight patients had one of the pre-defined acute surgical emergencies for inclusion in this study in 2019 (30.4%), and 94 in 2020 (23.1%). The mean age of patients presenting in 2019 was 41.2 years (± 21.7 years), versus 46.7 years (± 21.5 years) in 2020 (*P* = 0.034). Male to female ratio in 2019 was 0.64:1 (38.4% to 61.5%), *vs.* 0.69:1 (41.4% to 58.5%) in 2020 (*P* = 0.335) (Table 1).

**Table 1:** Demographics for acute emergency surgical presentations in March, April, and May 2019 versus the same time period in 2020

Demographics	2020	2019
Total number of surgical admissions to GUH (Mar–May)	406	453
March	88 (21.6%)	194 (42.8%)
April	153 (37.6%)	119 (26.2%)
May	165 (40.6%)	140 (30.9%)
Number of patients included in the study	94 (23.1%)	138 (30.4%)
Mean age ± SD (years)	41 (± 21.7)	47(± 21.5)
Gender		
Male	39 (41.4%)	53 (38.4%)
Female	55 (58.5%)	85 (61.5%)
Presentation by month (Total numbers)	94	138
March	30 (31.9%)	50 (36.2%)
April	30 (31.9%)	39 (28.2%)
May	34 (36.1%)	49 (35.5)
Presentation by specific pathology		
Acute cholecystitis	22	33
Acute appendicitis	43	78
Acute Diverticulitis	29	27

*GUH* Galway University Hospitals, *Mar* March, *SD* standard deviation

### Patterns of emergency surgical presentations

There was a 54.6% decrease in acute surgical admissions in March 2020 versus March 2019 (194 admissions in 2019 *vs.* 88 in 2020). A 22.2% increase in acute surgical admissions was observed between April 2020 *vs.* April 2019 (153 admissions in 2019 *vs.* 119 in 2020) (*P* = 0.748) (Table 2), as well as 17.9% increase in surgical admissions between May 2019 and May 2020 (140 admissions in May 2019 *vs.* 165 in May 2020).

**Table 2:** Cohort group presentation by month

Presentations	2020	2019
Cholecystitis		
Total	22	33
March	4 (18.1%)	15 (45.4%)
April	10 (45.4%)	11 (33.3%)
May	8 (36.3%)	7 (21.2%)
Appendicitis		
Total	43	78
March	17 (39.5%)	26 (33.3%)
April	12 (27.9%)	21 (26.9%)
May	14 (32.5%)	31 (39.7%)
Diverticulitis		
Total	29	27
March	9 (31.0%)	9 (33.3%)
April	8 (27.5%)	7 (25.9%)
May	12 (41.3%)	11 (40.7%)

**Figure. 1:**
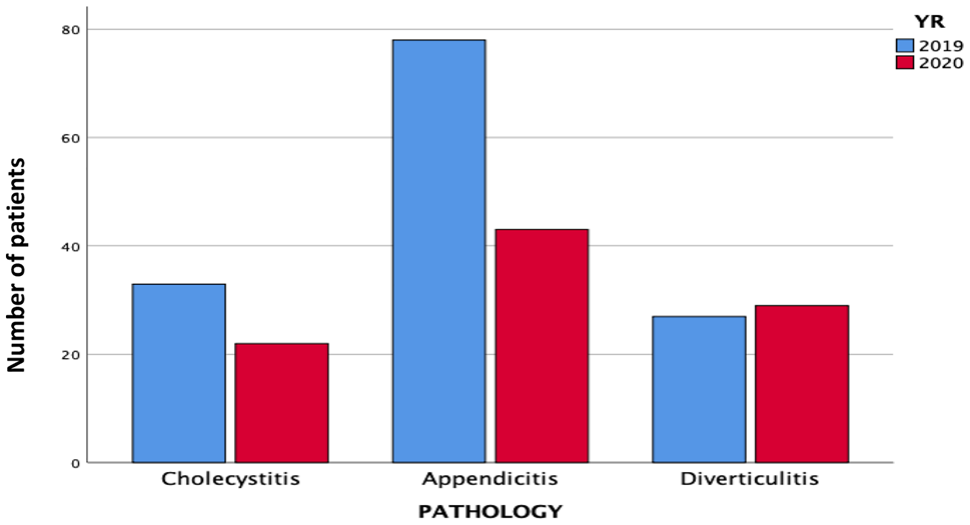
Pathology subgroup difference in presentations between 2020 and 2019

In the cholecystitis group, 15 patients presented with acute symptoms in March 2019, versus 4 patients in 2020, a decrease of 73.3%. This was followed by no reduction in April and May. In the appendicitis group, a significant reduction in acute presentations was observed between 2020 and 2019 for all months (34.6%, 42.8%, and 54.8% reductions for March, April, and May, respectively). In the diverticulitis group, similar number of presentations recorded in the 2 years with no significant difference throughout the study period (Table 2; Fig. 1).

### Duration of symptoms

The mean duration of symptoms prior to presenting to emergency surgical services was 2.70 (± 2.86) days in 2019 versus 3.13 (± 2.67) days in 2020 (*P* = 0.244). Subgroup analysis of the duration of symptoms for the three groups demonstrated no statistical significance. The largest difference was seen in the appendicitis group (*P* = 0.067), while the remaining two groups showed a similar pattern in both years (Table 3).

**Table 3:** Duration of symptoms

Year	2020	2019	*P-*value
Duration of symptoms (days)			
Cholecystitis	3.54	3.57	0.975
Appendicitis	2.4	1.8	0.067
Diverticulitis	3.7	4.1	0.708
Mean duration of symptoms	3.13 ± 2.67	2.70 ± 2.86	0.244

### Treatment

**Figure. 2:**
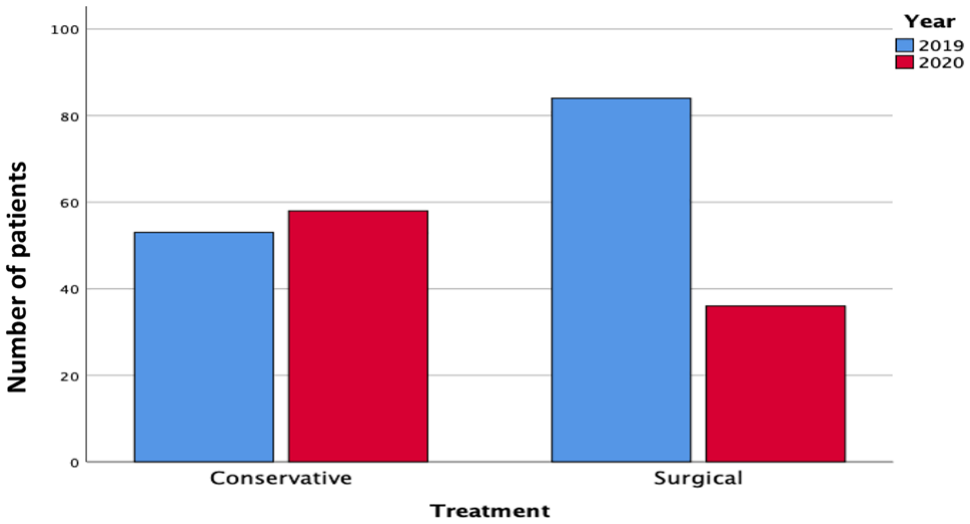
Overall treatment method adopted between 2020 and 201

**Figure. 3:**
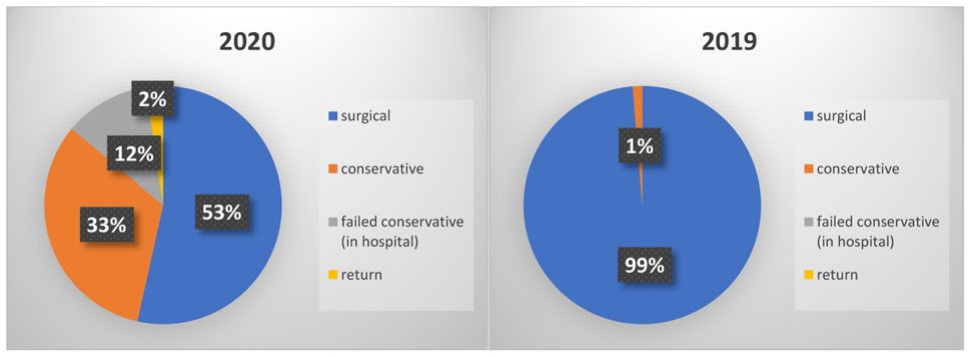
Management of acute presentations with appendicitis induring the study period

Fewer acute surgical procedures were performed in 2020 versus 2019. In 2019, 60.9% of patients admitted with acute surgical emergencies underwent surgical intervention (84/138), while 39.1% were treated conservatively (54/138) (Fig. 2). In 2020, 35.1% of admissions underwent surgical intervention (33/94), while 64.9% were treated conservatively (*P* < 0.001). The number of patients with operative management of acute appendicitis was lower in 2020 (29/43, 67.4%), compared to 2019 (77/78, 98.7%) (Fig. 3). Of the 20 patients initially treated conservatively for acute appendicitis (46.5%), 5 failed conservative management and proceeded to surgery (25%) (*P* < 0.001), while another patient represented within 30 days with recurrence of symptoms and underwent appendicectomy. Cross-sectional imaging (CT abdomen and pelvis) was utilised to aid diagnosis in 26 patients with appendicitis in 2020 (26/43, 60.4%) versus 13 in 2019 (13/78, 17.1%) (*P* = 0.001). One patient had a normal appendiceal histology following surgery in 2020 versus 7 in 2019 (1/43 *vs.* 7/78 *P* = 0.350) (Table 4).

**Table 4:** Breakdown of treatment method (conservative vs surgical)

Treatment	2020	2019	*P-*value
Total number of cases	94	138	
Surgical	33 (35.1%)	84 (60.8%)	< 0.001*
Conservative	61 (64.8%)	54 (39.1%)	
Cholecystitis	*N* = 22	*N* = 33	
Surgical	1	4	0.378
Conservative	21	29	
Appendicitis	*N* = 43	*N* = 78	
Surgical	29	77	< 0.001*
Conservative	14	1	
Diverticulitis	*N* = 29	*N* = 27	
Surgical	3	3	0.927
Conservative	26	24	

*N* number

^*^Statistical significance

### Morbidity and mortality

No major post-operative complications were recorded in either year of this study period for patients who underwent operative management of appendicitis, diverticulitis, or cholecystitis. In 2019, 77 patients with acute appendicitis were treated surgically (98.7%), *vs.* 29 in 2020 (67.4%). Of the 77 patients in 2019, 5 (6.5%) subsequently developed a post-operative complication, as did 7 of those treated in 2020 (24.1%) (*P* = 0.029). The incidence of post-operative complications was similar across both years for patients receiving surgical intervention for acute cholecystitis (*P* = 0.378). One patient died during this study (Table 5).

**Table 5:** Disease specific post-operative complications and mortality

	2020	2019	*P-*value
Cholecystitis	*N* = 1	*N* = 4	
None	0	0	
CD1	0	1	
CD2	1	3	*P* = 0.378
Mortality	0	1	
Appendicitis	*N* = 29	*N* = 76	
None	22	71	
CD1	4	3	
CD2	3	2	*P* = 0.029*
Diverticulitis	*N* = 3	*N* = 3	
None	0	3	
CD1	0	0	
CD2	1	0	

*CD* Clavien-Dindo morbidity scale, *N* number

^*^Statistical significance

### Length of hospital stay

There was an overall reduction in LOS from 2019 to 2020 (2019: 4.7 days *vs.* 2020: 4.3 day, *P* = 0.267). Median LOS was increased for patients with acute cholecystitis in 2020 compared to 2019 (6.5 days *vs.* 4 days, *P* = 0.606), and similarly was prolonged for patients admitted with appendicitis (2019: 2 days *vs.* 2020: 3 Days, *P* = 0.045). Decreased LOS was observed for patients with diverticulitis (2019: 4 days *vs.* 2020: 3 days) (*P* = 0.002) (Table 6).

**Table 6:** Total length of hospital-stay

	2020	2019	*P-*value
Median LOS (days)			
Cholecystitis	6.5 (0–25)	4 (0–60)	0.606
Appendicitis	3 (1–10)	2 (1–13)	0.045*
Diverticulitis	3 (0–14)	4 (2–18)	0.002*
Overall mean LOS	4.3	4.7	0.267

*LOS* length of hospital stay

^*^Statistical significance

### Readmissions

There was an overall increase in readmission rates in 2020 versus 2019 (11/94 *vs.* 7/138, *P* = 0.166). One patient from the cholecystitis group was readmitted within 30 days of discharge in 2019 (1/33), compared to 4 in 2020 (4/22, *P* = 0.056). Six patients with appendicitis were readmitted in 2019 (6/78) versus seven in 2020 (7/43, *P* = 0.326). There were no readmissions recorded for patients with diverticulitis in either year (Table 7).

**Table 7:** Thirty-day readmission rates for patients discharged with acute cholecystitis, appendicitis, and diverticulitis

Readmissions	2020	2019	*P-*value
Overall	11/94	7/138	0.166
Cholecystitis	4/22	1/33	0.056
Appendicitis	7/43	6/78	0.194
Diverticulitis	0/29	0/27	N/R

*N/R* not recordable

### Severity of symptoms and disease

The mean SAPS-II score of 13.92 ± 7.38 was recorded in 2019, compared to 16.25 ± 8.03 in 2020 (*P* = 0.031). Following subgroup analysis of SAPS-II score, an upward trend was noticed in all groups. The largest increase occurred in the cholecystitis group (2019: 20.0 ± 11.16 *vs.* 2020: 23.5 ± 10.2, *P* = 0.246), with the least in the appendicitis group (2019: 10.03 ± 4.04 *vs.* 2020: 10.5 ± 3.14, *P* = 0.487) (Table 8).

**Table 8:** Overall severity index at presentation by SAPS-II

Mean SPAS-II	2020	2019	*P-*value
Cholecystitis	23.5 (Std. 10.2)	20.0 (Std. 11.16)	0.246
Appendicitis	10.5 (Std 3.14)	10.03 (Std 4.04)	0.487
Diverticulitis	19.2 (Std. 6.19)	17.6 (Std 3.37)	0.258
Overall	16.25 (Std 8.03)	13.92 (7.38)	0.031

*SPAS-11* Simplified Acute Physiology Score II, *std* standard deviation

An increase in disease severity was noted among patients presenting with cholecystitis in 2020, with a mean severity grade of 1.6 in 2019 compared to 2.0 in 2020, based on the Tokyo Guidelines classification (*P* = 0.079) (Table 9). There was an increase in the number of patients presenting with severe acute cholecystitis, with 7/22 (31.8%) presenting with grade 3 disease compared to 3/33 patients in 2019 (9.0%, *P* = 0.043). For patients with acute appendicitis, the mean APSI score was 4.5 (± 3.33) in 2020 *vs.* 2.1 (± 1.16) in 2019 (*P* < 0.001) (Table 9). In 2020, the mean DSS score was 1.75 (± 1.214) *vs.* 1.35 (± 0.817) in 2019 (*P* = 0.003) (Table 9). For patients with diverticulitis, similar grades of disease were observed each year using the Hinchey classification system to stratify disease severity (*P* = 0.930) (Table 9).

**Table 9:** Disease specific severity score

	2020	2019	*P* value
TG18/13	(*N* = 22)	(*N* = 33)	
G1	8	15	
G2	6	15	
G3	7	3	
Mean	2.0	1.6	0.079
APSI	(*N* = 26)	(*N* = 13)	
0	1	1	
1	10	3	
2	5	2	
3	5	4	
4	5	2	
5	1	1	
Mean	4.5 (Std. 3.33)	2.1 (Std 1.16)	< 0.001
DSS	*N* = 29	*N* = 77	
Mean	1.75(Std 1.214)	1.35 (Std 0.817)	0.003
Hinchey	*N* = 29	*N* = 27	
1	23	18	
2	6	9	
3	0	0	0.930

*N* number, *TG* Tokyo Guidelines 18/13, *G* grade, *APSI* radiological appendicitis severity index, *Std* standard deviation, *DSS* disease severity score

## Discussion

The outbreak of the COVID-19 pandemic has forced healthcare systems across the globe to adapt practices, allocate scarce resources, and tailor management strategies in order to optimize patient care. Our understanding of COVID-19 and the threat posed by novel variants remains uncertain, and there is currently a paucity of evidence pertaining to the pathophysiological, natural history, and molecular pathways of COVID-19, with surgical guidelines and management protocols relating to COVID-19 continuing to evolve rapidly. Therefore, the aim of the present study was to quantitatively analyse differences in presentation, management, and outcomes of acute surgical conditions during the initial months of the COVID-19 pandemic compared with the previous year. The most significant finding in this analysis of almost 900 acute surgical presentations was the decline in presentations to the emergency department with acute surgical pathology, as well as the increased severity of symptomology during the COVID-19 pandemic.

These results reflect an extremely worrying trend hypothesised by expert consultant surgeons worldwide; during the initial phase of the COVID-19 pandemic, symptomatic patients requiring emergency surgical admission elected to isolate and suffer at home, instead of presenting to hospital, given the potential implications of COVID-19. This hypothesis is supported by the data captured in this study, with an overall declining trend in acute surgical presentations of 31.8%, including a staggering 54.6% decline during March. Furthermore, patients presented with more severe subjective symptomology, and had increased hospital LOS. A similar trend was observed during the SARS pandemic, when a Taiwanese study demonstrated widespread patient reluctance to attend medical services, due to a perceived risk of viral transmission and infection [13]. Moreover, the Orsola-Malpighi University Hospital in Bologna, Italy, have reported a greater than 70% decrease in ocular emergency presentations during COVID-19 [3], while large pooled data from 47 American states outline a 20% reduction in acute stroke presentations as well as a 10% decrease in hyperglycaemic crisis during the initial phase of the COVID-19 pandemic [2].

Associated with the reduced number of presentations, increased severity of disease, and overall increased LOS, patients also reported experiencing symptoms for a longer duration prior to presenting to hospital; the mean duration of symptoms was 2.70 days in 2019 *vs.* 3.13 days in 2020. Once more, this illustrates a worrying trend affecting the delivery of care to surgical patients. This appears to be translatable to other medical specialties, as data presented by Queen Mary Hospital, Hong Kong, demonstrated a four-fold increase in the time between onset of symptoms in patients presenting with acute myocardial infarction to first medical contact [6]. An Italian study incorporating 5 paediatric hospitals over 1 week demonstrated 12 cases of delayed presentations, of which 6 required an immediate admission for intensive care, and mortality occurred in 4 children [4]. It is well established that for patients with acute surgical pathology requiring emergency surgery, the timing of presentation from initiation of symptoms is an important factor in determining disease severity and patient outcomes [14, 15]. Thus, the authors wish to reiterate the serious implications of delayed presentations on clinical outcomes for patients of all demographics and in various healthcare settings, and highlight the barrier presented by COVID-19 as physicians worldwide attempt to provide benchmark care.

Results from this analysis highlight excellent true-appendicitis rates during the COVID-era; surgeons correctly diagnosed appendicitis in 97.7% of cases in patients who had an appendicectomy in 2020, compared to in 91.0% of cases in 2019. The authors acknowledge that this statistic presents a mere consolation when we acknowledge the increased subjective symptomology, reduced number of presentations, and significant reduction in patients managed operatively for this acute pathology. Increased utilisation of CT imaging to aid clinical decision making is likely to have affected the overall success rate in acute appendicitis detection. In well-resourced healthcare systems, perhaps incorporation of stringent imaging modalities into acute surgical assessment pathways may reduce the overall occurrence of false appendicitis rates, which have reported to be has high as 20% in some series [16].

The current global pandemic has had a number of implications which continue to limit surgical practice. Staff redeployment, requirement to reserve hospitals beds for COVID positive patients, scarcity of personal protective equipment, risk of viral transmission among care givers, and uncertainty surrounding the impact of possible infection on patients post-operatively have all placed restraints on global surgery, as described by the COVIDSurg collaborative, among others [17–23]. Surgical services were necessitated to adapt their approach toward conservative measures where possible, as outlined in this analysis, with intervention limited to urgent time-sensitive malignancies and life-threatening emergency surgery in the setting of the critically unwell.

This study has obvious limitations, being a retrospective cohort study, conducted in a single centre. Details pertaining to clinical outcomes are limited as a consequence of this; however, remain pertinent in detailing how COVID-19 has impacted surgical services at our tertiary referral centre.

This study highlights the manner in which COVID-19 has reduced the number of acute emergency surgical presentations during the initial 3-month period of the pandemic, as patients present with subjectively severe symptoms following an initial delay. Patient education surrounding the serious nature and potentially catastrophic impact of acute surgical emergencies seems imperative, as we continuously adapt surgical practice in response to the many challenges which threaten the delivery of excellent patient care during the current global public health crisis.
